# Neuro-oncological Ventral Antigen 2 Regulates Splicing of Vascular Endothelial Growth Factor Receptor 1 and Is Required for Endothelial Function

**DOI:** 10.1007/s43032-022-01044-4

**Published:** 2022-08-04

**Authors:** Veerle Kremer, Jetta J. Oppelaar, Theresa Gimbel, Susanne Koziarek, Wessel Ganzevoort, Mariëlle G. van Pampus, Bert-Jan van den Born, Liffert Vogt, Christianne de Groot, Reinier A. Boon

**Affiliations:** 1 Department of Physiology, Amsterdam Cardiovascular Sciences, VU Medical Center, Amsterdam UMC, Amsterdam, The Netherlands; 2 Department of Medical Chemistry, Academic Medical Center, Amsterdam UMC, Amsterdam, The Netherlands; 3 Amsterdam Cardiovascular Sciences, Microcirculation, Amsterdam, The Netherlands; 4 Department of Internal Medicine, Section of Nephrology, Amsterdam UMC Location University of Amsterdam, Meibergdreef 9, Amsterdam, The Netherlands; 5 Institute of Cardiovascular Regeneration, Goethe University, Frankfurt am Main, Germany; 6 German Centre for Cardiovascular Research DZHK, Partner Site Frankfurt Rhein/Main, Frankfurt am Main, Germany; 7 Department of Obstetrics and Gynecology, Amsterdam Reproduction & Development, Amsterdam UMC University of Amsterdam, Amsterdam, The Netherlands; 8 Department of Obstetrics and Gynecology, OLVG Hospital, Amsterdam, The Netherlands; 9 Department of Internal Medicine, Section of Vascular Medicine, Amsterdam UMC Location University of Amsterdam, Meibergdreef 9, Amsterdam, The Netherlands; 10 Amsterdam Cardiovascular Sciences, Atherosclerosis and Ischemic Syndromes, Amsterdam, The Netherlands; 11 Department of Obstetrics and Gynaecology, Amsterdam UMC, Vrije Universiteit, Amsterdam, The Netherlands; 12 Amsterdam UMC, De Boelelaan 1108, 1081 HZ Amsterdam, The Netherlands

**Keywords:** Pre-eclampsia, Placenta, Endothelium, Alternative Splicing, FLT1

## Abstract

Pre-eclampsia (PE) affects 2–8% of pregnancies and is responsible for significant morbidity and mortality. The maternal clinical syndrome (defined by hypertension, proteinuria, and organ dysfunction) is the result of endothelial dysfunction. The endothelial response to increased levels of soluble FMS-like Tyrosine Kinase 1 (sFLT1) is thought to play a central role. sFLT1 is released from multiple tissues and binds VEGF with high affinity and antagonizes VEGF. Expression of soluble variants of *sFLT1* is a result of alternative splicing; however, the mechanism is incompletely understood. We hypothesize that neuro-oncological ventral antigen 2 (NOVA2) contributes to this. *NOVA2* was inhibited in human umbilical vein endothelial cells (HUVECs) and multiple cellular functions were assessed. *NOVA2* and *FLT1* expression in the placenta of PE, pregnancy-induced hypertension, and normotensive controls was measured by RT-qPCR. Loss of *NOVA2* in HUVECs resulted in significantly increased levels of *sFLT1*, but did not affect expression of membrane-bound *FLT1*. NOVA2 protein was shown to directly interact with *FLT1* mRNA. Loss of *NOVA2* was also accompanied by impaired endothelial functions such as sprouting. We were able to restore sprouting capacity by exogenous VEGF. We did not observe statistically significant regulation of *NOVA2* or *sFLT1* in the placenta. However, we observed a negative correlation between *sFLT1* and *NOVA2* expression levels. In conclusion, NOVA2 was found to regulate *FLT1* splicing in the endothelium. Loss of *NOVA2* resulted in impaired endothelial function, at least partially dependent on VEGF. In PE patients, we observed a negative correlation between *NOVA2* and *sFLT1*.

**Supplementary Information:**

The online version contains supplementary material available at 10.1007/s43032-022-01044-4.

## Introduction

Pre-eclampsia (PE) is a complication affecting 2–8% of pregnancies and is an important contributor to morbidity and mortality [1]. PE is thought to be the result of a two-stage process in which placental oxidative stress and inflammation lead to the release of soluble factors such as pro-inflammatory cytokines and anti-angiogenic factors. The imbalance in angiogenic factors then leads to systemic maternal endothelial disease and the clinical symptoms that are the result of increased vascular reactivity and impaired endothelial barrier integrity (high blood pressure, proteinuria, and organ dysfunction) [1–4]. One of the key angiogenic factors in endothelial cells (EC) is vascular endothelial growth factor (VEGF) which binds and activates VEGF-receptor 1 (FLT1), VEGF-receptor 2 (VEGFR2), or Neuropilin-1. VEGFR2 binds VEGF with lower affinity, but its intracellular domain has high tyrosine kinase activity which induces EC migration and proliferation [5, 6]. FLT1 binds VEGFA as well as placental growth factor (PlGF). In comparison to VEGFR2, FLT1 has higher affinity for VEGFA but weak tyrosine kinase activity [6–8]. The *FLT1* mRNA transcript contains 30 exons and is alternatively spliced to give rise to membrane-bound (*mFLT1*) and soluble (*sFLT1*) isoforms, which lack the intracellular domain [4]. *sFLT1-i13* and *sFLT1-e15a* are the most abundant sFLT1 splice forms [9, 10]. *sFLT1-i13* forms when splicing of exons 13 to 14 is skipped and the transcript reads through into intron 13 and uses an alternative polyadenylation signal in intron 13. In case of *sFLT1-e15a*, there is alternative splicing of exon 15 [11]. sFLT1 acts as a potent antagonist of VEGF and PlGF since it binds with high affinity but lacks a signalling domain [12, 13]. PE is characterized by increased sFLT1 levels in placenta and circulation [14, 15] as well as increased mRNA expression of *sFLT1* variants [16–18]. However, increased sFLT1 expression was not always accompanied by increased mFLT1 expression [15, 16]. This would suggest that in the development of PE there is no change in transcription of precursor *FLT1* mRNA, rather there is a shift in alternative splicing to favor production of *sFLT1* rather than full-length *mFLT1*.

Neuro-oncological ventral antigen 2 (NOVA2) was first characterized as an immunoreactive protein in paraneoplastic opsoclonus myoclonus ataxia l. NOVA2 shares 85% amino acid homology with NOVA1 and both contain K homology (KH) RNA binding domains. NOVA2 is expressed throughout the brain and was found to be crucial for axon pathfinding during development. Nova2 null mice failed to thrive and showed motor dysfunction or weakness and died after approximately 18 days [19, 20]. NOVA2 regulates pre-mRNA splicing by binding directly to a cluster of intronic YCAY motifs [21]. The binding position of NOVA2 determines the outcome of the splicing event. Generally, binding to clusters of intronic YCAY motif upstream of the alternative exon promotes exon skipping. NOVA2 interaction with the intronic region downstream of the alternative exon promoted exon inclusion [22, 23]. A second mechanism through which NOVA2 can regulate expression of different gene transcripts is alternative polyadenylation (APA). A large proportion of genes contains more than one polyadenylation site and multiple transcript isoforms can be generated with alternative 3′ ends. The use of different polyadenylation sites can affect RNA stability or localization. The majority of APA sites are in the 3′ UTR but other APA sites can induce premature polyadenylation within the coding region [24]. Nova2 binds YCAY motifs flanking polyadenylation sites and the position of Nova2 binding determines whether a polyadenylation site is used or not [25, 26]. In addition to the brain, NOVA2 was also found to be expressed in the endothelium and is crucial for vascular development and lumen formation in zebrafish [27].

The regulation of *FLT1* splicing is not fully known and we aim to better understand how splicing of this transcript is regulated. We hypothesize that splicing factor NOVA2 contributes to *FLT1* splicing in the endothelium since NOVA2 is known to play a role in EC function in mice and zebrafish. Furthermore, our goal is to characterize the role of NOVA2 both in endothelial function and PE.

## Materials and Methods

### Cell Pellets

Pelleted cells of different cell types in the cardiovascular system were purchased from Promocell. RNA was isolated using miRNeasy micro kit (Qiagen). Sequencing was performed on the NextSeq500 instrument (Illumina) using v2 chemistry and 1 × 75 bp single-end setup as described previously [28]. Trimmed and filtered reads were aligned versus the Ensembl human genome version hg38 (GRCh38) using STAR 2.6.1d with the parameter “–outFilterMismatchNoverLmax 0.1” to increase the maximum ratio of mismatches to mapped length to 10%. The number of reads aligning to genes was counted with featureCounts 1.6.5 from the Subread package.

### Sequencing of Mouse Hearts

To validate our in vitro sequencing in tissue, cardiomyocytes and non-cardiomyocytes were isolated from 8-week-old male wild-type C57BL/6 mice as described previously by Trembinski et al. [29]. Briefly, cardiomyocytes were separated from other cells, such as fibroblasts and ECs, via density centrifugation. RNA isolation was done using the RNeasy Mini Kit and the RNase-Free DNase Set (Qiagen). RNA sequencing was performed as described previously [30]. Briefly, poly-A RNA was selected using poly-T oligo attached beads. Sequencing libraries were prepared using the TruSeq RNA sample preparation kit (Illumina) and sequencing was done using the HiSeq 2000 flowcell (Illumina). Reads were mapped using TopHat (2 mismatches) and gene expression was estimated using Cufflinks version 2.1 with default parameters.

### Cell Culture

Human umbilical vein endothelial cells (HUVECs) were purchased from Lonza (lots p1028 and p1032) and cultured in endothelial cell medium (ECM) supplemented with endothelial cell growth supplement (ECGS), penicillin/streptomycin (P/S), and 5% FBS (all Sciencell). Cells were cultured at 37 °C and 5% CO_2_. Cells between passages 1 and 4 were used for experiments. Cells were counted using the Countess II Automated Cell Counter (Thermo Fisher Scientific).

### Transfection

HUVECs were transfected at 60–80% confluence with 50-nM siRNA (Sigma Aldrich) using Lipofectamine RNAiMax (Thermo Fisher Scientific) in OptiMEM Glutamax (Gibco). As a control, non-targeting siRNA (Sigma Aldrich) was transfected. After 4 h, the medium was replaced with full ECM. Oligo sequences can be found in Table s2.

### RNA Isolation and RT-qPCR

Total RNA was isolated from cells using TRIzol (Thermo Fisher Scientific) and the Direct-zol RNA miniprep kit (Zymo Research) according to the manufacturer’s instructions including DNase I digestion. For RNA isolation from placenta, lysis was done using the TissueLyser II (Qiagen). RNA was isolated using the Direct-zol RNA miniprep kit including DNase I digestion. For cDNA synthesis, 1000 ng of total RNA was reverse transcribed using oligo(dT) and random primers (iScript cDNA synthesis kit, BioRad). RT-qPCR was performed using iQ SYBR Green Supermix (BioRad) in a CFX96 or CFX384 Touch Real-Time PCR Detection System (BioRad). Gene expression analysis was done using the 2^−dCt^ method. Primers can be found in Table s2.

### Spheroid Assay

After 24-h transfection, HUVECs were trypsinized and resuspended in ECM culture medium containing 0.6 g/L methylcellulose (Sigma Aldrich). Spheroids were formed by seeding HUVECs (400 cells per well in 100 μL) in U-bottom 96-well plates (Costar) and culturing for 24 h at 37 °C and 5% CO_2_. Spheroids were resuspended in FBS (Sciencell) containing 2.4 g/L methylcellulose (dissolved in ECM) and mixed 1:1 with collagen-I solution (3.77 g/L collagen-I (Corning, USA), 10% M199 medium (Sigma Aldrich), 0.018 M HEPES, and 0.2 M NaOH to adjust pH to 7.4). In a 24-well plate, 1 mL of the mixture was added per well and polymerized for 30 min at 37 °C. In total, 100 μL of ECM with or without VEGF (50 ng/mL, Preprotech) was added. Spheroids were fixed after 24 h using 10% formaldehyde in PBS and visualized using a Zeiss DMIL LED microscope. The cumulative sprout length per spheroid was measured using ImageJ software.

### Endothelial Barrier Function Assay

Endothelial barrier was measured using electrical cell-substrate impedance sensing (ECIS, Applied Biophysics). Cells were seeded 24 h after transfection at a density of 100,000 cells/well in 350 μL full ECM. Prior to seeding, electrodes of the 8W10E plate (Ibidi) were coated with 10 mM l-cysteine (Sigma Aldrich) and 1% gelatin (Merck). Resistance was measured at the multi-frequency setting.

### Proliferation Assay

After 20 h of transfection, cell proliferation was measured using the Click-iT EdU cell proliferation kit (Thermo Fisher Scientific) in 8-well μ-slides (Ibidi) at a density of 30,000 cells/well. EdU (final concentration 10 μM) was added to each well and fixation and staining was done after 24-h EdU incubation. Nuclei were stained using Hoechst. Cells were imaged using the ZOE fluorescent cell imager (BioRad). Total cell number and EdU positive cells were counted.

### Western Blot

HUVECs were lysed 48 h after transfection in radioimmunoprecipitation assay (RIPA) buffer (Sigma Aldrich) with protease and phosphatase inhibitors (Halt inhibitor cocktail, Thermo Fisher Scientific). Protein concentration was measured using Pierce BCA protein assay kit (Thermo Fisher Scientific). A total of 10 μg of protein was separated on SDS-PAGE gels, transferred to nitrocellulose membranes, blocked in block buffer (TBST + 5% BSA + 0.05% sodium azide) for 1 h, and incubated overnight at 4 °C with primary antibodies. Secondary antibodies (Dako) were incubated for 2 h at room temperature in TBST + 5% BSA. Bands were visualized using enhanced chemiluminescence (ECL, Amersham/GE-Healthcare) on the AI600 (Amersham/GE-Healthcare). Original full-length blots can be found in Supplemental Fig. 2. Antibodies and dilutions can be found in Table s2.

### Apoptosis

Caspase-3/7 activity was measured as a marker of apoptosis using the Apo ONE Homogenous Caspase-3/7 assay (Promega) according to the manufacturer’s instructions. HUVECs were seeded in a 96-well plate 45 h after transfection. Staurosporine (Sigma Aldrich) was added to adhered cells at a final concentration of 200 nM. After 4 h, caspase reagent was diluted in caspase buffer and added to each well. Fluorescence was measured at excitation/emission 485/521 nm using the FLUOstar Galaxy (BMG).

### Cross-linking RNA Immunoprecipitation (CLIP)

CLIP was performed as described previously [31]. RNA enrichment was quantified by RT-pPCR. cDNA was synthesized as described previously. Alternatively, after incubation of beads with the lysate, beads were washed, resuspended in 5 × sample buffer (312.5 mM Tris, pH 6.8, 50% glycerol, 0.37 mM bromophenol blue, 10% SDS, 2.5% β-mercaptoethanol) to be analyzed by Western blot. Primers, antibodies, and concentrations can be found in Table s2.

### Patients

Patients were included as part of the Non-Osmotic Sodium storage In Placental tissue (NOSIP) study, a multicenter prospective case–control study to investigate the role of sodium accumulation in the placenta in relation to maternal and fetal outcomes in women with hypertensive disorders of pregnancy. Subjects included in this analysis have provided separate written informed consent to participate in the NOSIP biobank which aims to investigate how the placenta contributes to hypertension in pregnancy. The study was conducted at the Amsterdam UMC and the OLVG Oost, after approval of the ethics committee of the Academic Medical Center on March 22, 2019 (METC 2019_013). Before the first inclusion, the study was registered at The Netherlands Trial Register (NL7640). All subjects provided written informed consent and the studies were conducted in accordance with the Declaration of Helsinki. Placenta tissue was stored after delivery at 4 °C until sectioning. After removal of the maternal and fetal membranes, full-thickness biopsies of 1 cm^2^ were taken around the insertion of the umbilical cord. Sections were stored fresh frozen at – 80 °C until RNA isolation.

### ELISA

Of the NOSIP study participants, blood was collected within 72 h before labor. Plasma samples were stored at − 80 °C before processing. Concentrations of sFLT1 and PlGF were measured by ELISA according to the manufacturer’s instructions. Samples were diluted 3 times prior to use and measured in duplicate. The following kits were used: sFLT1 (myBiosource, MBS2601616) and PlGF (R&D, PDPG00).

### Statistical Analysis

Data is presented as mean ± standard error of the mean (SEM). D’Agostino-Pearson test was used to assess departure from normality/normal distribution. Between-groups comparisons were made using a *t*-test or Mann–Whitney test. When comparing more than two groups, ANOVA or Kruskal–Wallis test was performed including Holm-Sidak or Dunn’s correction for multiple testing. For in vitro experiments, we have performed paired experiments since we have run each experiment at least 3 times, each time with a control and treated sample handled in parallel. When appropriate, we therefore performed paired analysis comparing each treatment with its own control, within individual experiments. GraphPad Prism 9 (GraphPad Software, San Diego, USA) was used for the analysis. A *p*-value < 0.05 was considered statistically significant.

## Results

*NOVA2* Silencing Results in Induction of *sFLT1* But Not *mFLT1* mRNA Levels.

**Fig. 1:**
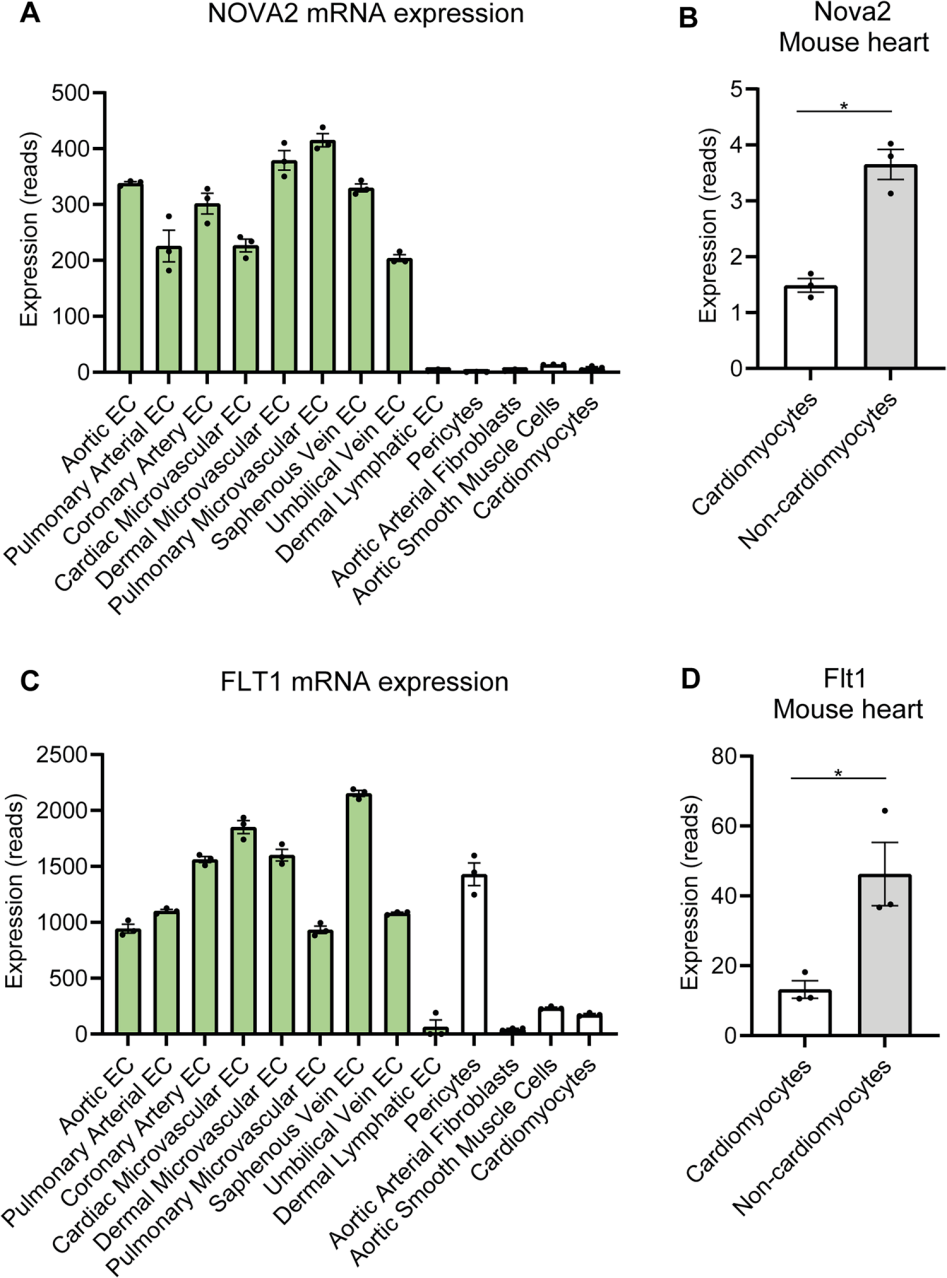
NOVA2 and FLT1 are enriched in ECs. **A, C** We obtained pelleted cells of different cell types in the cardiovascular system from Promocell and RNA sequencing was performed using different cell types from the cardiovascular system. **A** *NOVA2* and **C** *FLT1* are shown. **B, D** RNA was isolated from mouse hearts. Cardiomyocytes were separated from other cells by density centrifugation and used for RNA sequencing. **B** *Nova2* and **D** *Flt1* are shown. Groups were analyzed using an unpaired *t*-test. Three samples were included in each group. Data are presented as mean ± SEM. Significance was indicated as follows: *p < 0.05, **p < 0.01, ***p < 0.001, not significant (ns)

**Fig. 2:**
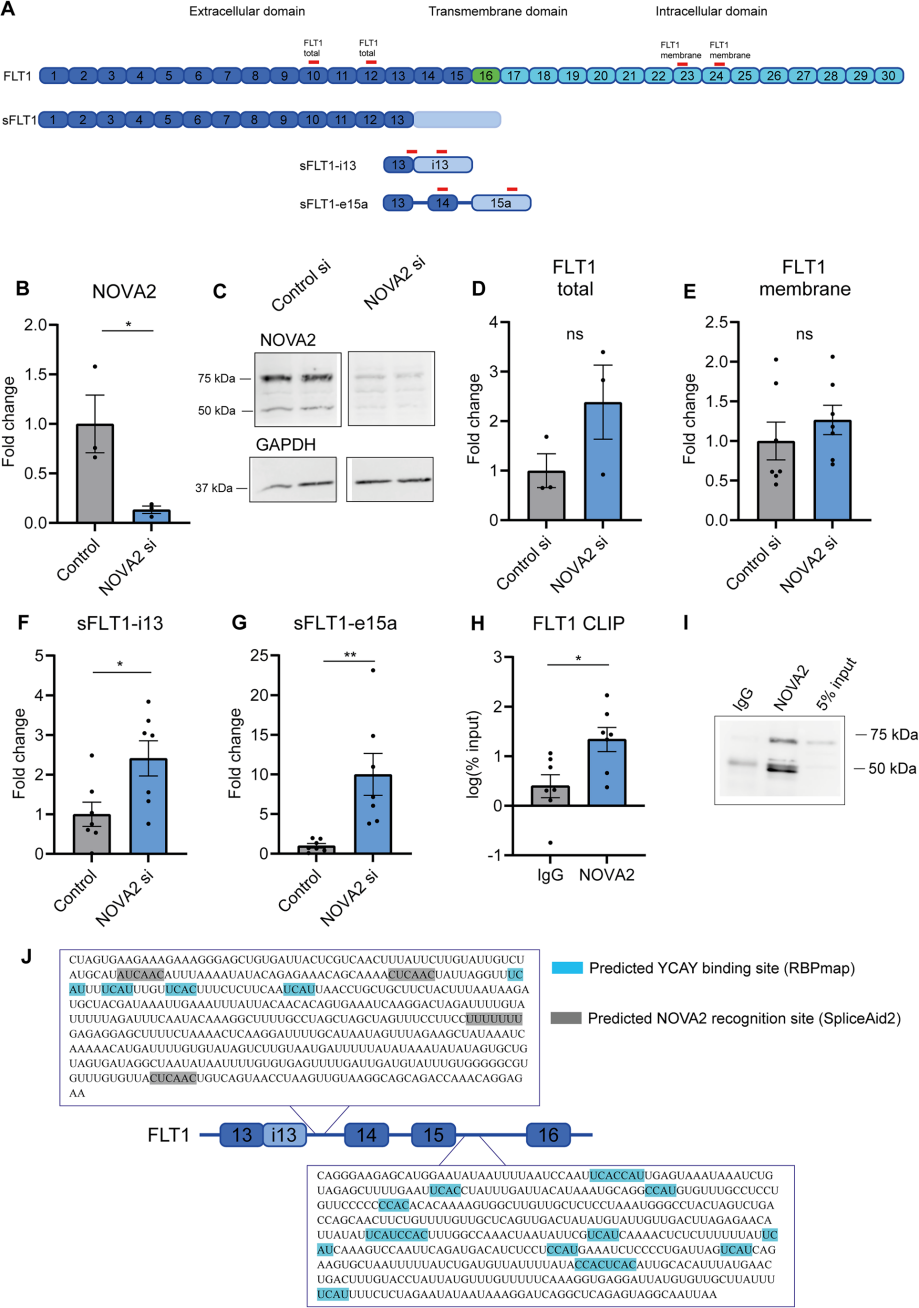
Induction of sFLT1 but not FLT1 mRNA levels following NOVA2 silencing in HUVECs. **A** Schematic representation of FLT1 variants and binding site of RT-qPCR primers. **B** NOVA2 was silenced in HUVECs using siRNA. A non-targeting siRNA was transfected as a control. RNA was isolated 48 h after transfection and *NOVA2* expression was measured by RT-qPCR. Expression is normalized to *RPLP0*. Groups were compared using a paired *t*-test. 3 independent experiments were performed. **C** NOVA2 protein levels were determined using Western blot. Cell lysates were collected 48 h after transfection. GAPDH was used as a loading control. Images were cropped and contrast was increased for clarity. 2 experiments were performed. **D–G** NOVA2 was silenced using siRNA. A non-targeting siRNA was transfected as a control. RNA was isolated 48 h after transfection and **D** total *FLT1*, **E** membrane *FLT1*, **F** *sFLT1-i13*, and **G** *sFLT1-e15a* expression was measured by RT-qPCR. Expression is normalized to *RPLP0*. Groups were compared using a paired *t*-test. 3–7 independent experiments were performed. **H, I** *FLT1* binding to NOVA2 was analyzed in HUVECs by RT-qPCR following CLIP. Non-targeting IgG was used as a control. Enrichment was quantified relative to input. Groups were compared using a paired *t*-test. 6 independent experiments were performed. **I** NOVA2 pulldown was detected using Western blot. Image was cropped and contrast was increased for clarity. Of note, the IgG heavy chain detected at 50 kDa overlaps with the NOVA2 band. **J** Schematic representation of human *FLT1*. Exons are in blue; the transcript reads into intron 13 for *sFLT1-i13* are in light blue. The highlighted YCAY sites are identified by RBPmap and SpliceAid2 within 500 nt from the exon–intron junction. For each potential binding site predicted by RBPmap, the *Z*-score > 2.52 and *p* < 5.57e–03. Data are presented as mean ± SEM. Significance was indicated as follows: *p < 0.05, **p < 0.01, ***p < 0.001, not significant (ns)

To assess the distribution of *NOVA2* expression in the vascular system, we performed RNA sequencing using commercially bought cells of different types in the cardiovascular system. We observed enrichment of *NOVA2* in venous, arterial, and microvascular endothelial cells (Fig. 1A). In addition, sequencing data from The Human Protein Atlas showed *NOVA2* is highly expressed in the brain, lung, and placenta [32]. In order to assess whether EC enrichment is also observed in tissue, and is not an artifact of our in vitro setup, RNA sequencing was also performed using cells isolated from the mouse heart. We observed an increase in *Nova2* expression in non-cardiomyocytes compared to cardiomyocytes (Fig. 1B). RNA sequencing also indicated EC enrichment of *FLT1*, compared to fibroblasts and cardiomyocytes (Fig. 1C). In the mouse, *FLT1* was significantly higher expressed in non-cardiomyocytes compared to cardiomyocytes (Fig. 1D). Data from the Human Protein Atlas suggested *FLT1* is enriched in the placenta, more specifically in trophoblasts as well as ECs. Since NOVA2 is a known regulator of splicing, we were interested to see whether NOVA2 is involved in splicing of FLT1. In a HUVEC model, siRNA-mediated knockdown reduced NOVA2 at both mRNA and protein level (Fig. 2B, C; Supplemental Fig. 1A). Loss of *NOVA2* using siRNA 1 did not significantly affect *mFLT1* mRNA expression (Fig. 2A, D, E). There was an increase in mFLT1 using siRNA 2 and 3. We observed a statistically significant induction of 2 sFLT1 variants, namely *sFLT1-i13* and *sFLT1-e15a* (Fig. 2F, G; Supplemental Fig.1B). The induction of *sFLT-e15a* was most pronounced. Furthermore, CLIP showed significant *FLT1* mRNA binding to NOVA2, suggesting NOVA2 can bind *FLT1* to affect its splicing (Fig. 2H, I). NOVA2 binds clusters of YCAY sequences, generally defined as > 3 YCAY sites within 45 nucleotides [33]. We used RBPmap [34] and SpliceAid2 [35] prediction tools to look for potential NOVA2 binding sites in the *FLT1* transcript (Fig. 2J). We observed multiple potential NOVA2 binding sites in the introns downstream of exon 13 and 15 and we hypothesize that NOVA2 binding to these regions results in exon inclusion and a shift towards full-length FLT1.

Loss of *NOVA2* Results in Mild Barrier Impairment and Diminishes Sprouting Under Basal VEGF Levels.

**Fig. 3:**
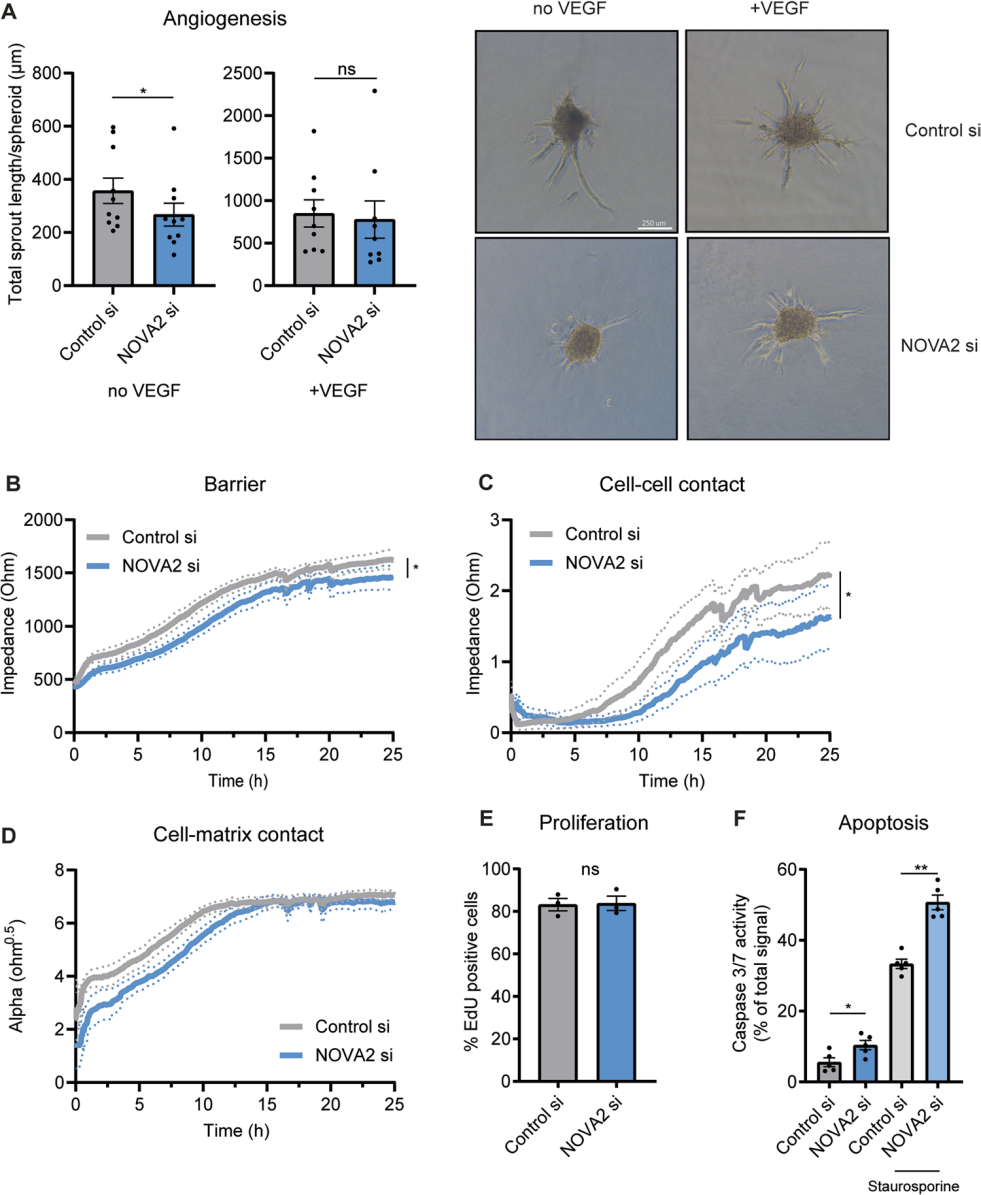
Loss of NOVA2S results in impaired sprouting and mild barrier impairment. **A** EC spheroids were embedded in collagen gels 24 h after transfection and stimulated with VEGF. Fixation was done after 24-h VEGF stimulation. Cumulative sprout length was determined by measuring the distance from the base of the spheroid to the tip cell. Discontinuous sprouts were excluded. 10 experiments were performed; for each independent experiment, 7–12 spheroids were scored in each experiment. Scale bar indicates 250 μm. **B–D** HUVECs were seeded 24 h after transfection at a density of 100,000 cells/well in 8W10E ECIS plates. Impedance was measured continuously. By altering the frequency, overall barrier (**B**), cell–cell contact (**C**), and cell–matrix contact (**D**) can be distinguished. Groups were analyzed at *t* = 24 h using paired *t*-test. 7 independent experiments were performed. **E** Proliferation was measured by EdU incorporation between 24 and 48 h after transfection. The percentage of proliferating cells is shown. Groups were analyzed using a paired *t*-test. 3 experiments were performed. **F** HUVECs were seeded in a 96-well plate 45 h after transfection. Apoptosis was induced using 200-nm staurosporine. Caspase substrate was added and fluorescence was measured after 1 h. Fluorescence intensity for each sample was normalized to the total signal from the plate. Groups were analyzed using a paired one-way ANOVA. 4 experiments were performed. Data are presented as mean ± SEM. Significance was indicated as follows: *p < 0.05, **p < 0.01, ***p < 0.001, not significant (ns)

We measured phosphorylation of ERK1/2 as a measure of downstream VEGF signalling since FLT1 was known to inhibit angiogenesis through VEGF capture [12] (Supplemental Fig. 1C). ERK1/2 phosphorylation was low under basal conditions and there was a strong induction upon exogenous VEGF stimulation, both under control or si-NOVA2 conditions. These observations suggest that downstream VEGF signalling in response to high exogenous VEGF levels is not affected upon *NOVA2* depletion. To better understand the effect of NOVA2 loss under physiological VEGF levels, we assessed angiogenic sprouting in vitro. Loss of *NOVA2* resulted in reduced sprouting under basal conditions (Fig. 3A). Stimulation of sprouting using exogenous VEGF rescued sprouting in *NOVA2* silenced cells. We next used the ECIS setup to assess endothelial barrier. Loss of *NOVA2* resulted in mildly impaired endothelial barrier after 24 h (Fig. 3B). Furthermore, cell–cell contacts were affected (Fig. 3C). There appeared to be no change in cell–matrix connections (Fig. 3D). Multiple processes are involved in forming new vessels, such as proliferation and migration. To better understand how NOVA2 regulates sprouting, proliferation was assessed in NOVA2-silenced HUVECs. We observed no differences between control and *NOVA2* knockdown (Fig. 3E). There was increased cell death as assessed by caspase 3/7 activity under basal conditions (Fig. 3F).

Reduced *sFLT1/mFLT1* Ratio Correlates with Increased *NOVA2* Expression.

**Fig. 4:**
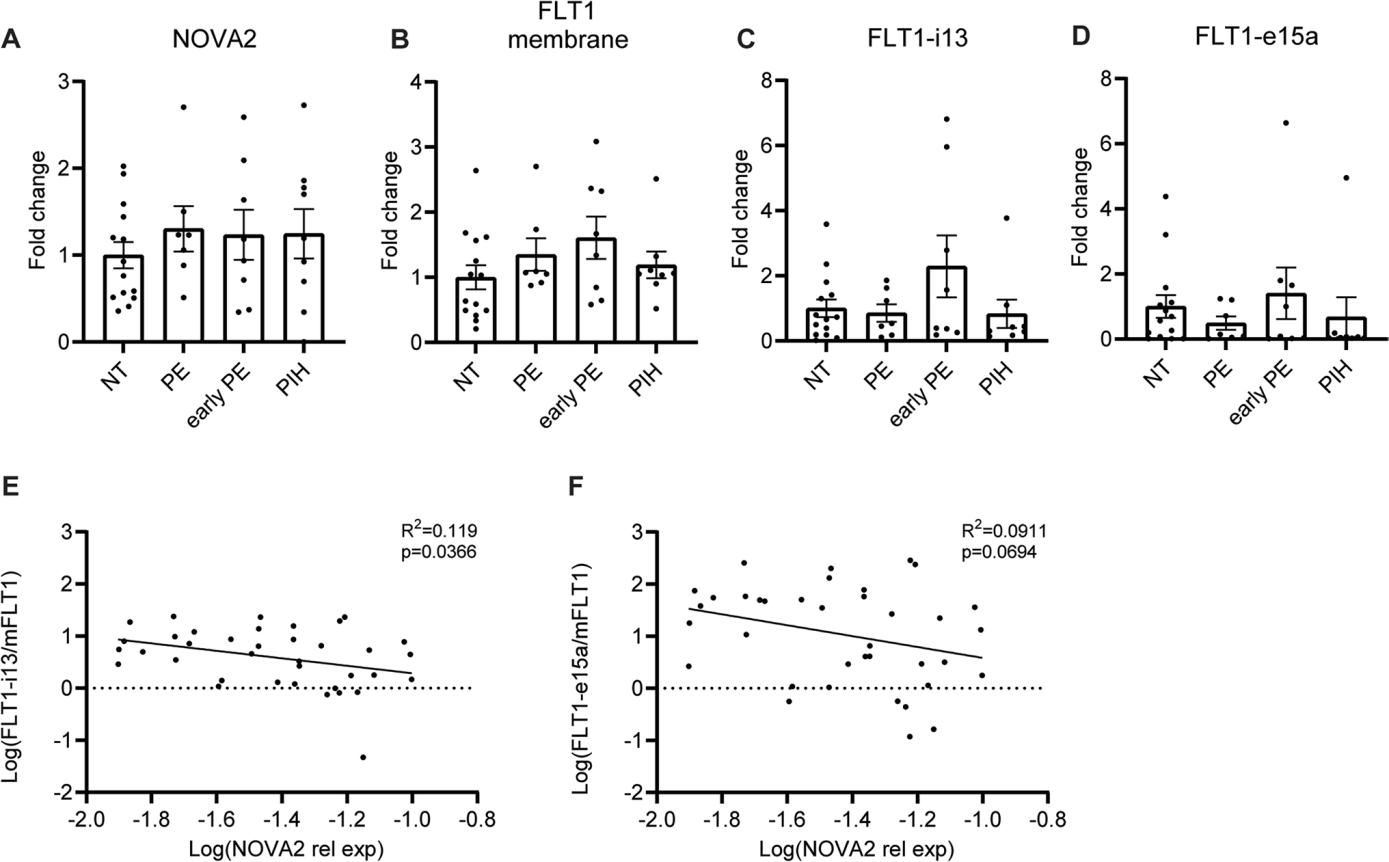
Reduced sFLT1/mFLT1 ratio correlates with increased NOVA2 expression. **A**–**D** Frozen placenta tissue was obtained from 15 PE, 8 PIH patients, and 14 NT controls. RNA was isolated and **A** *NOVA2*, **B** *mFLT1*, **C** *sFLT1-i13*, and **D** *sFLT1-e15a* were measured by RT-qPCR. Expression is normalized to *SDHA*. Groups were compared using one-way ANOVA. **E**, **F** *sFLT1* relative expression correlation with *NOVA2* relative expression. Correlation was estimated using simple linear regression. Data are presented as mean ± SEM. Significance was indicated as follows: *p < 0.05, **p < 0.01, ***p < 0.001, not significant (ns)

Excess sFLT1 is thought to contribute significantly to EC dysfunction which results in clinical symptoms of pre-eclampsia [17] and we investigated a potential role for NOVA2 in this process. Placenta tissue and blood were collected from 37 patients diagnosed with PE (early and late onset) and pregnancy-induced hypertension (PIH) patients as well as normotensive (NT) controls. Systolic and diastolic blood pressure was increased in PE and PIH patients compared to NT controls and the placenta was smaller on average (Table s1). Early PE patients delivered at a lower gestational age. We observed only a very mild upregulation of sFLT1 in the circulation in PE and PIH patients (Supplemental Fig. 1D). In addition, there was a reduction in PlGF levels in these patients (Supplemental Fig. 1E). We observed a significant increase in the sFLT1:PlGF ratio in PE patients compared to NT (Supplemental Fig. 1F), which is consistent with previous findings in PE patients [36]. In our study, plasma levels of PlGF and especially sFLT1 were relatively low compared to other studies [14, 37]. We observed no statistically significant changes in *NOVA2* (Fig. 4A) or *FLT1* (Fig. 4B-D) mRNA expression. We investigated a potential correlation between *NOVA2* and *sFLT1*. First, we normalized *sFLT1* to membrane *FLT1* levels since there was high variation in expression levels between patients. Moreover, we were interested to investigate the effect of NOVA2 on *FLT1* splicing specifically. There was a weak but statistically significant negative correlation between the *sFLT1-i13*:*mFLT1* ratio and *NOVA2* mRNA and a trend towards a negative correlation between *sFLT1-e15a:mFLT1* and *NOVA2* (Fig. 4E, F).

## Discussion

In this study, we highlight a previously undefined role for NOVA2 in the regulation of *FLT1* splicing. We demonstrate that loss of NOVA2 is accompanied by increased *sFLT1* expression, while NOVA2 interacts with *FLT1* mRNA. *NOVA2* depletion resulted in impaired EC function such as sprouting, which is thought to be at least in part mediated by sFLT1 since VEGF stimulation ameliorated the effect of *NOVA2* knockdown. Also, endothelial barrier function was impaired and there was increased apoptosis. In the placenta of normotensive controls and PE patients, we observed a negative correlation between *sFLT1* and *NOVA2* expression levels. Previous research has indicated that NOVA2 is upregulated at both mRNA and protein levels in HUVECs exposed to hypoxia [38]. NOVA2 was also found to be upregulated specifically in ECs in several cancers such as colorectal [38] and ovarian cancer [39]. In addition to upregulation of NOVA2 under hypoxia, hypoxia downregulates *sFLT1* but not *mFLT1* mRNA in human microvascular ECs (HMVEC). The exact mechanism was not known, but it has been suggested that RNA binding proteins are involved which regulate alternative splicing of *FLT1* [40] and several proteins have indeed been described which potentially regulate splicing of *FLT1*. Overexpression of hnRNP D resulted in decreased *sFLT1* mRNA in HMVECs. There was also a change in *mFLT1* levels, although this was less pronounced [41]. JMJD6 together with U2AF65 binds to *FLT1* and is also thought to regulate its splicing in HUVECs [42], although Eddy et al. suggested that the role of JMJD6 and U2AF655 in *FLT1* splicing may be limited and that there may be differences between cell types [43]. The principal sources of FLT1 in the placenta are trophoblasts, ECs, and macrophages [10, 44]. Soluble variant *sFLT1-e15a* specifically is mainly produced by the syncytiotrophoblast and cytotrophoblast, and to a lesser extent by ECs, vascular smooth muscle cells, and macrophages [10]. *sFLT1-i13* expression was detected in vascular endothelial cells by RNA in situ hybridization [45]. Upon knockdown of *NOVA2*, we observed an increase in not only *sFLT1* but also *mFLT1* levels, which would suggest that NOVA2 can contribute to *FLT1* transcription as well as splicing. This effect on transcription could be an indirect effect. NOVA2 can regulate alternative splicing of transcription factors or their binding partners, which in turn results in altered expression of target genes such as *FLT1*. Belloni et al. show that Nova2 can regulate expression levels of key genes in EC function in mice such as *Flt1*, *Flt4*, and *Eng*. More specifically, Nova2 regulated alternative splicing of *Tfdp2* which increased Tfdp2-E2F1–mediated transcription [22]. In addition to splicing, NOVA2 could also play a role in mRNA localization [46] or mRNA stability through nonsense-mediated decay [47] and based on our data we cannot exclude whether these effects play a role as well. Taken together, this would suggest a multi-layered function of NOVA2 in the endothelium. The increase in *sFLT1-i13* levels could be due in part to increased *FLT1* transcription. The increase in *sFLT1-e15a* was more pronounced than could be expected based on an increase in transcription and is more likely due to a change in splicing. To confirm this, CLIP showed NOVA2 interacting with *FLT1*, which would suggest NOVA2 could mediate *FLT1* splicing. It was previously shown that NOVA2 binding downstream of an exon would promote its inclusion [23]. We hypothesize that NOVA2 binding downstream of exon 13 or 15 promotes inclusion of exons 14–30 and the production of full-length *FLT1*. To better understand downstream VEGFR signalling, we investigated phosphorylation of ERK1/2. There was an increase in phosphorylation upon VEGF stimulation, both under control and si-NOVA2 conditions. This suggests downstream VEGF signalling is not affected by NOVA2. This effect could be cell type specific. For example, in human lymphatic EC, there was sustained ERK1/2 phosphorylation in NOVA2-deficient cells stimulated with VEGFC. In control cells, pERK1/2 levels diminished more quickly. The authors did not find global dysregulation of signalling after loss of NOVA2, but rather a specific regulation of ERK1/2 signalling by NOVA2 [48]. Since loss of NOVA2 is accompanied by increased levels of *sFLT1*, we hypothesize that there would be impaired EC function after NOVA2 knockdown. Loss of NOVA2 in HUVECs was associated with impaired sprouting under unstimulated conditions. Adding exogenous VEGF improved sprouting in NOVA2 silenced cells. These data suggest that the induction of sFLT1 can contribute to impaired sprouting. NOVA2 is known to regulate splicing of multiple genes in EC, shown in mouse ECs by Giampietro et al. [27]. Gene ontology clustering showed enrichment for genes involved in cell adhesion and cytoskeletal remodelling, which could play an important role in angiogenesis. Therefore, it is possible that NOVA2 exhibits additional functions in the endothelium independent of FLT1. Furthermore, here was an increase in caspase 3/7 activity after loss of NOVA2. In accordance with this, Zhai et al. observed an increase in apoptosis in HUVECs stimulated with sFLT1 [49]; however, we cannot exclude that NOVA2 also regulates apoptosis independent of sFLT1. NOVA2 was previously shown to play a role in barrier maintenance in different vascular beds in human and mouse. Giampietro et al. observed increased permeability to FITC-dextran upon Nova2 knockdown in mouse ECs isolated from the lung [27]. This would suggest that macromolecular permeation is affected by NOVA2. Low expression NOVA2 was also shown to increase blood–brain barrier permeability. In this study, NOVA2 was shown to induce expression of tight junction proteins such as ZO-1, occludin, and claudin-5 [50]. Using the ECIS system, we can further differentiate between basal adhesion of the cells (shown as cell–matrix connections) as well as paracellular flux (shown as cell–cell connections) [51]. In our HUVEC model, loss of NOVA2 resulted in overall impairment of the barrier. A decrease in cell–cell connections was the most important contributor to this. Thus, induction of *sFLT1* after loss of NOVA results in endothelial cell impairment, likely through inhibition of low-grade VEGF signalling, which can be overcome by exogenous VEGF. There are several strengths and limitations to consider for our studies. The use of an in vitro EC model allows specific depletion of NOVA2 to better understand its function. Since both *FLT1* and *NOVA2* are highly expressed in EC compared to other cell types in the vasculature, this suggests HUVECs are a very relevant model. *NOVA2* and *FLT1* are both expressed in HUVECs; however, an important limitation of this model is the fact that HUVECs are isolated from the umbilical vein of newborns, and are therefore not the best representation of the vascular bed as is found in adults. Within our group of patients, the gestational age was similar for NT, late PE, and PIH patients, but early PE patients delivered earlier on average. This should be kept in mind since FLT1 and PlGF levels vary throughout pregnancy [14]. We did not observe a statistically significant change in *sFLT1* expression whereas previous research had demonstrated increased *sFLT1* mRNA levels [17]. Also, in the blood samples collected from PE patients, levels of PlGF and especially sFLT1 in circulation were lower than expected based on previous studies [14, 37, 52]. It is important to note that the sample size was rather limited, and therefore we might not be able to detect statistically significant changes. It is also possible that large changes in *sFLT1* expression, and perhaps also *NOVA2*, occur earlier in the onset of the disease. This would be in accordance with the first stage of the two-stage model of PE, when anti-angiogenic factors are released from the placenta following insufficient perfusion [2]. Our findings do suggest that changes in *sFLT1* and *NOVA2* negatively correlate at the mRNA level, although the correlation is rather weak as indicated by the *R*^2^ value of 0.119. Our studies cannot show whether NOVA2 is also regulated at the post-transcriptional level during PE rather than at the mRNA level. Treatment strategies for PE such as antithrombin are currently under investigation. Another innovation is the use of siRNAs to silence *sFLT1* expression. Turanov et al. showed reduced *sFLT1* expression and improvement of clinical symptoms in a baboon PE model treated with sFLT1 siRNA [53–55]. Modulation of NOVA2 could potentially be another tool to limit sFLT1 production in the placenta. The findings in this study do not exclude that NOVA2 could also mediate effects in the endothelium through a mechanism independent of *FLT1* splicing which could further reduce endothelial dysfunction and ameliorate maternal syndrome. In order to translate our findings to the clinic, it is crucial to also understand the role of NOVA2 in the development of the placenta as well as fetal development. It should also be kept in mind that any placental lesion which is driving the development of PE is likely still present after excessive sFLT1 production is inhibited and could affect fetal development.

In conclusion, NOVA2 was found to regulate splicing of *FLT1* in the endothelium. Loss of NOVA2 resulted in impaired endothelial function. Exogenous VEGF ameliorates the angiogenesis inhibition in NOVA2-depleted cells. These observations suggest that the increase in *sFLT* causally contributes to the angiogenesis impairment seen after loss of NOVA2. In PE patients, we observed a negative correlation between *NOVA2* and *sFLT1*, suggesting NOVA2 could contribute to the imbalance in angiogenic factors in PE.

## Supplementary Information

Below is the link to the electronic supplementary material.

Supplementary file1 (TIF 16235 KB)

Supplementary file2 (TIF 25835 KB)

Supplementary file3 (TIF 8089 KB)

Supplementary file4 (TIF 22198 KB)

## Data Availability

Available upon request.
