# Supplementary figures and images for: Neuro-oncological Ventral Antigen 2 Regulates Splicing of Vascular Endothelial Growth Factor Receptor 1 and Is Required for Endothelial Function
Source: Reprod Sci. 2022 Aug 4;30(2):678–89. doi: 10.1007/s43032-022-01044-4 (PMC9988812; doi:10.1007/s43032-022-01044-4)

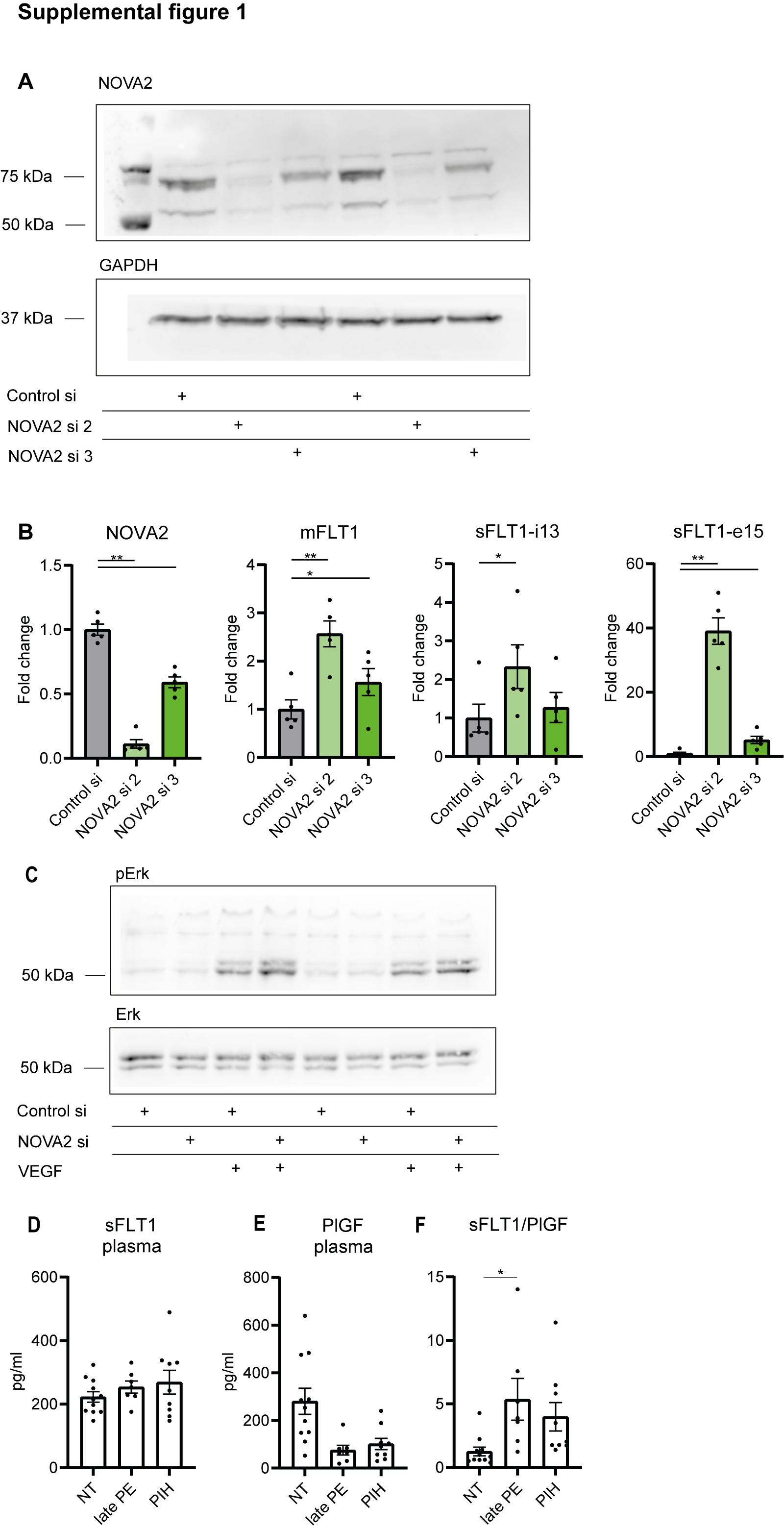

Supplement: Supplementary file 1 — Supplementary file1 (TIF 16235 KB) [file 43032_2022_1044_MOESM1_ESM.tif]

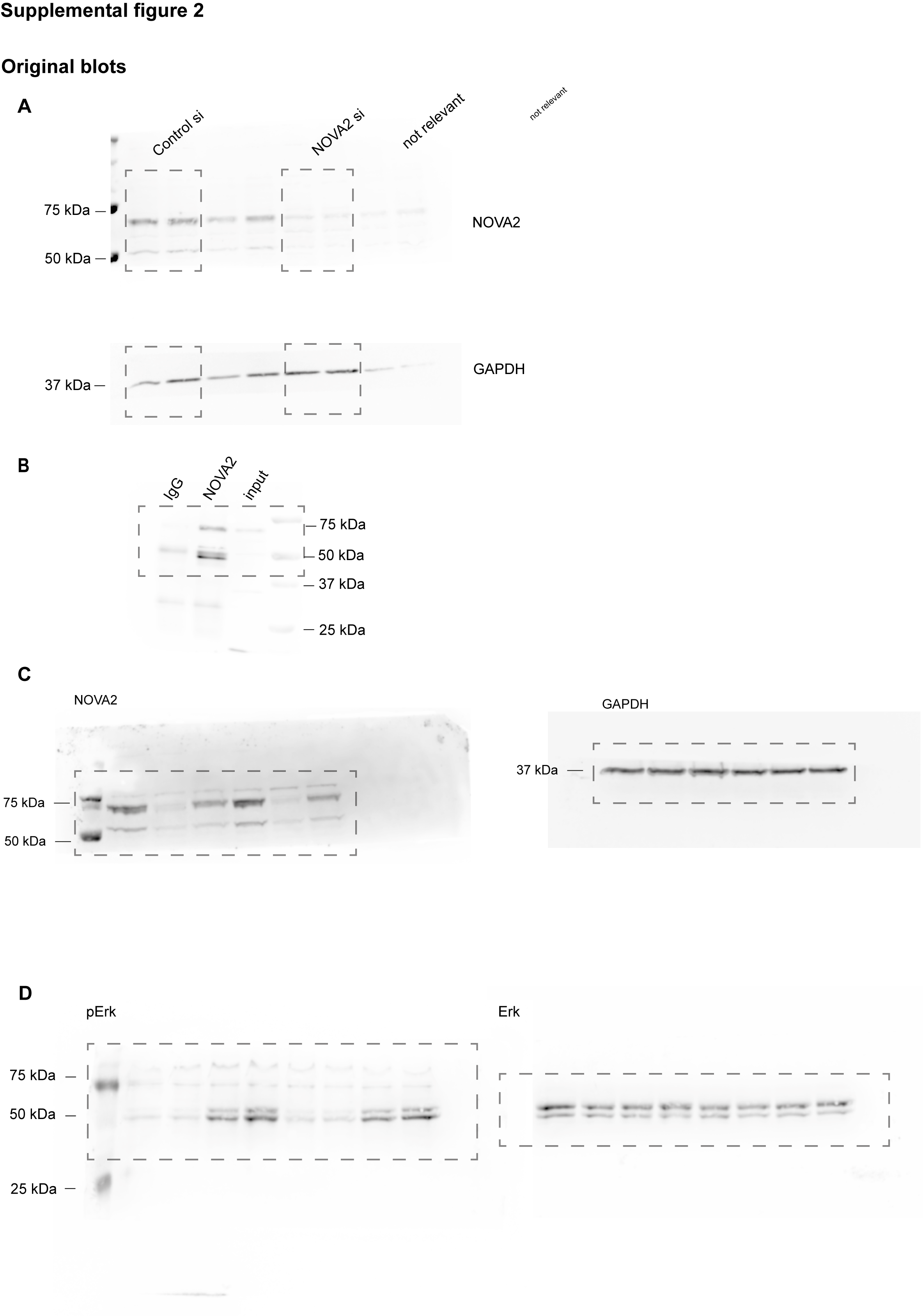

Supplement: Supplementary file 2 — Supplementary file2 (TIF 25835 KB) [file 43032_2022_1044_MOESM2_ESM.tif]

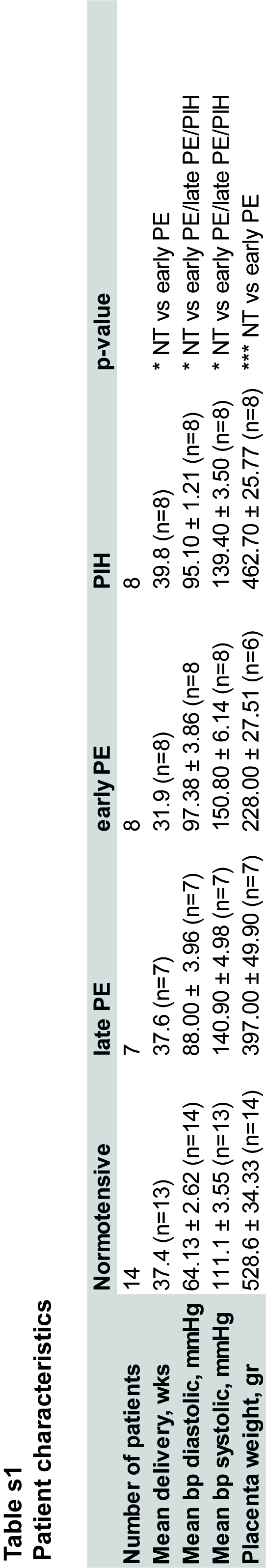

Supplement: Supplementary file 3 — Supplementary file3 (TIF 8089 KB) [file 43032_2022_1044_MOESM3_ESM.tif]

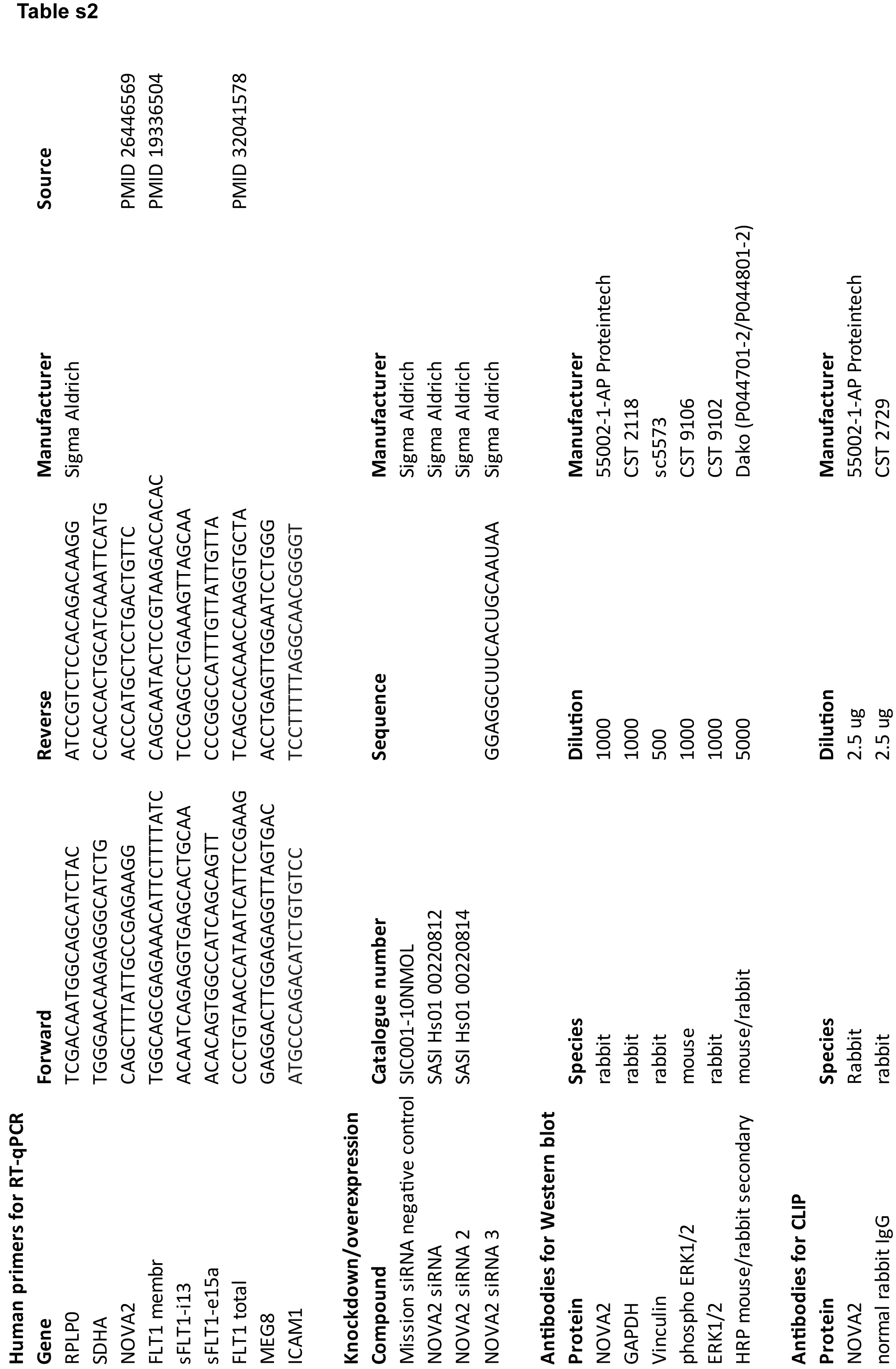

Supplement: Supplementary file 4 — Supplementary file4 (TIF 22198 KB) [file 43032_2022_1044_MOESM4_ESM.tif]
